# Dysuria due to discospondylitis and intervertebral disc herniation in a male alpaca (*Vicugna pacos*)

**DOI:** 10.1186/s13028-016-0216-5

**Published:** 2016-05-31

**Authors:** Marlene Sickinger, Manuela Hirz, Martin J. Schmidt, Manfred Reinacher

**Affiliations:** 1 Clinic for Ruminants, Justus-Liebig-University of Giessen, Frankfurter Str. 110, 35392 Giessen, Germany; 2 Institute of Veterinary Pathology, Justus-Liebig-University of Giessen, Frankfurter Str. 96, 35392 Giessen, Germany; 3 Clinic for Small Animals (Department of Surgery), Justus-Liebig-University of Giessen, Frankfurter Str. 108, 35392 Giessen, Germany; 4 Clinic for Obstetrics, Gynecology and Andrology with Veterinary Ambulance, Justus-Liebig-University of Giessen, Frankfurter Str. 106, 35392 Giessen, Germany

**Keywords:** Alpaca, *Vicugna pacos*, Discospondylitis, Disc herniation, Dysuria

## Abstract

**Background:**

Dysuria in camelids is usually associated with the presence of lower urinary tract disease such as urolithiasis. As another differential diagnosis, urine retention may be caused by neurological disturbances resulting from infections of the spinal cord, discospondylitis or trauma.

**Case presentation:**

A 2.5-year-old male Huacaya alpaca (*Vicugna pacos*) presented with dysuria due to damage of the lumbosacral intumescence of the spinal cord. On presentation the alpaca was recumbent. Clinical examination revealed abdominal pain, oliguria, leucopenia with neutrophilia, and slightly elevated creatinine kinase. Ultrasonography of the abdomen showed an irregularly shaped, dilated urinary bladder with hyperechoic serosa. Magnetic resonance imaging revealed discospondylitis of the fourth and fifth lumbar vertebrae and herniation of the intervertebral disc between these vertebrae and the spinal cord. Postmortem examination confirmed severe chronic purulent discospondylitis with ventral spondylosis and narrowing of the spinal canal. Urolithiasis could not be verified.

**Conclusion:**

Although rare, diseases of the spinal cord should be considered as a differential diagnosis for impaired micturition in camelids.

## Background

Ischuria in camelids is most commonly caused by urethral obstruction attributable to urolithiasis [1, 2]. Spinal diseases, such as lesions of the lower motor neurons (LMN) or upper motor neurons (UMN), resulting from discospondylitis, epidural abscesses, or trauma have been reported to cause urine retention in cats and dogs [3]; however, this has not been reported in new world camelids.

## Case presentation

A 2.5-yr-old male Huacaya alpaca presented with reduced food intake, slight abdominal distension, straining, and abdominal pain. The animal had been recumbent for 24 h. At the time of presentation, the alpaca was slightly lethargic but seemed to be in good physical condition (body weight, 68 kg). Feces were of normal consistency, with its color ranging from light olive to orange. Abdominal tension was palpated using the positive ballottement test. Urine output was subjectively reduced and cystocentesis was performed. Urinalysis revealed leucocytes, bacteria, glucose, protein, and amorphous phosphate crystals, and cystitis was subsequently diagnosed. Hematology and chemistry panel revealed leucopenia of 4.6 g/l (range, 8–21.4 g/l) with neutrophilia (3.5 g/l neutrophil granulocytes, 0.75 g/l lymphocytes, 0.3 g/l monocytes, and 0.05 g/l eosinophil granulocytes). Sodium, potassium, and ionized calcium concentrations as well as urea and creatinine concentrations were within reference ranges. Creatinine kinase was elevated to 740 U/l (range, 25–200 U/l).

Abdominal ultrasound was performed with a Mindray M5 using a 5-MHz convex transducer (Sonoring Holzwickede, Germany). A bilateral ventrolateral approach was used. Gastric compartments and small and large intestinal loops were identified and assessed. No structural abnormalities (normal intestinal peristalsis and no accumulation of peritoneal effusion) were noted. Ultrasonography of the kidneys and urinary bladder revealed bilateral, mild pyelectasia and dilation of the urinary bladder, which had an irregular shape and hyperechoic serosa. Small floating hyperechoic contents were visible after careful agitation of the urinary bladder. Due to rapid progression of colic symptoms, the decision was made to perform a tube cystotomy. The alpaca was anesthetized with xylazine (Albrecht, Aulendorf, Germany; 0.15 mg/kg intravenous (IV)) and ketamine (CP Pharma, Burgdorf, Germany; 4 mg/kg IV) and prepared for surgery. The animal was intubated, and anesthesia was maintained with inhaled 1.5 % isoflurane carried in oxygen (2 l/min; Albrecht). A right paramedian laparotomy was performed for trocarization of the urinary bladder and placement of a balloon catheter (supracath^®^ puncture set, CH 16, Teleflex, Athlone, Ireland) to achieve drainage of the organ. The serosa of the urinary bladder was hyperemic, with distended blood vessels on its surface. However, uroliths were not observed.

Perioperative medical treatment consisted of scopolamine butylbromid and metamizol-sodium as antispasmodics and analgesics (Boehringer Ingelheim, Ingelheim, Germany; 0.3 and 37 mg/kg twice daily IV for 3 days), benzylpenicillin-sodium as antimicrobial treatment (belapharm, Vechta, Germany; 30,000 IU/kg once daily IV for 3 days), and dexamethasone to prevent fibrinous adhesions (belapharm; 0.12 mg/kg once daily IV for 2 days). The day after surgery the alpaca showed no signs of colic but remained recumbent.

Two days after tube cystotomy, pelvic limb ataxia developed. The neurologic examination revealed ambulatory paraparesis with absent proprioception of the hind limbs. When lifted, the alpaca was able to stand for a few seconds but proprioception was absent. Cutaneous trunci reflex was reduced caudal to the fifth lumbar vertebra (L5), with superficial and deep pain being highly reduced for both hind limbs. Withdrawal reflex of the pelvic limbs was slightly reduced bilaterally. Tibialis cranialis and flexor reflex for both hind limbs as well as the perineal reflex were reduced. Examination of cranial nerves revealed no abnormal reactions and behavior was unaffected. Based on clinical examination, a spinal cord lesion was suspected on L4-S5.

**Fig. 1:**
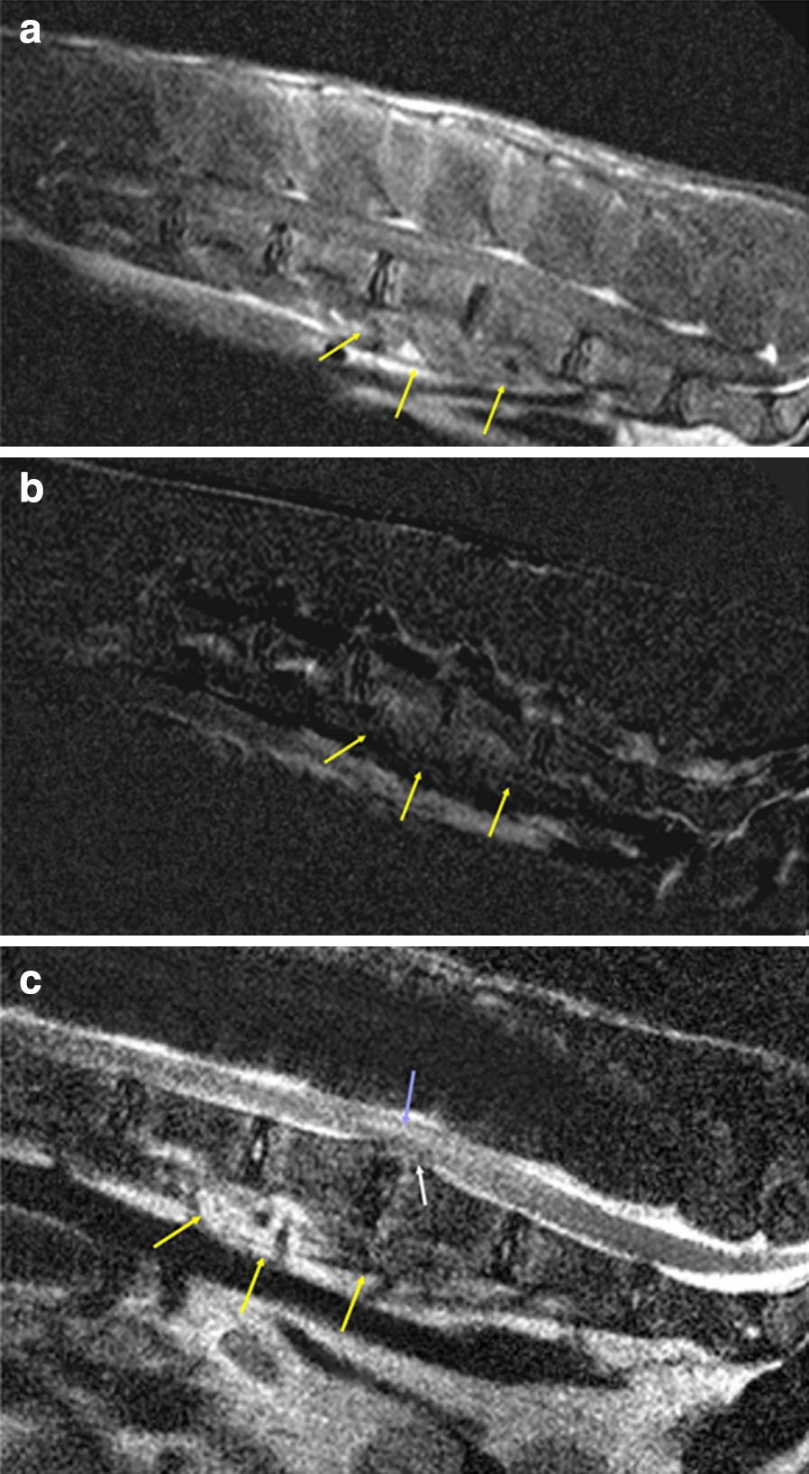
Magnetic resonance images of the lumbar spine of a male alpaca (*Vicugna pacos*). **a** T1-weighted sagittal MRI, **b** subtraction image and **c** T2-weighted sagittal MRI of the lumbar spine of a male alpaca. There is moderate enhancement of the endplates of L4/5, the ventral epidural space, and the sublumbar muscles. The endplates between the fourth and fifth lumbar vertebrae show irregular margins and the intervertebral disc space is *narrowed*. The intervertebral disc is degenerated and protrudes dorsally, leading to spinal cord compression. The myelon shows an irregular area of high signal intensity above the disc material

The alpaca was anesthetized as previously described and magnetic resonance imaging (MRI) was performed (Fig. 1). The magnetic resonance (MR) image displayed diffuse irregular endplates between L4 and L5, with almost total loss of the nucleus pulposus signal of the corresponding disc. There was mild subluxation between the two vertebrae and moderate bulging of the disc, leading to compression of the adjacent spinal cord. The myelon itself showed an irregular area of high signal intensity above the disc material extending rostrally and caudally to the end of L4 and L5. Ventral to the two vertebrae, the hypaxial musculature also revealed a heterogeneously hyperintense signal extending to the dorsal epaxial muscles. After administration of contrast medium (Dotarem^®^, Guerbet, Zürich, Switzerland; 0.2 ml/kg IV) there was moderate enhancement of the endplates of L4/L5, the ventral epidural space, and the sublumbar muscles. Based on these findings, a tentative diagnosis of discospondylitis with herniation of the corresponding disc, cellulitis of the epidural fat, and spinal cord compression with intramedullary edema was made. In addition, the inflammatory process had spread to the surrounding tissue, leading to myositis.

Decompression of the spinal cord and removal of the inflamed tissue were recommended. The owner declined surgical treatment and the animal was humanely euthanized.

Gross postmortem examination revealed a pale, yellow, viscous mass within the intervertebral space and involving the intervertebral disc between L4 and L5. Additionally, the ventral sides of vertebral bodies L4 and L5 were connected to each other by a bridging bone formation (spondylosis). Urolithiasis was not present and the urinary bladder showed submucosal hyperemia.

**Fig. 2:**
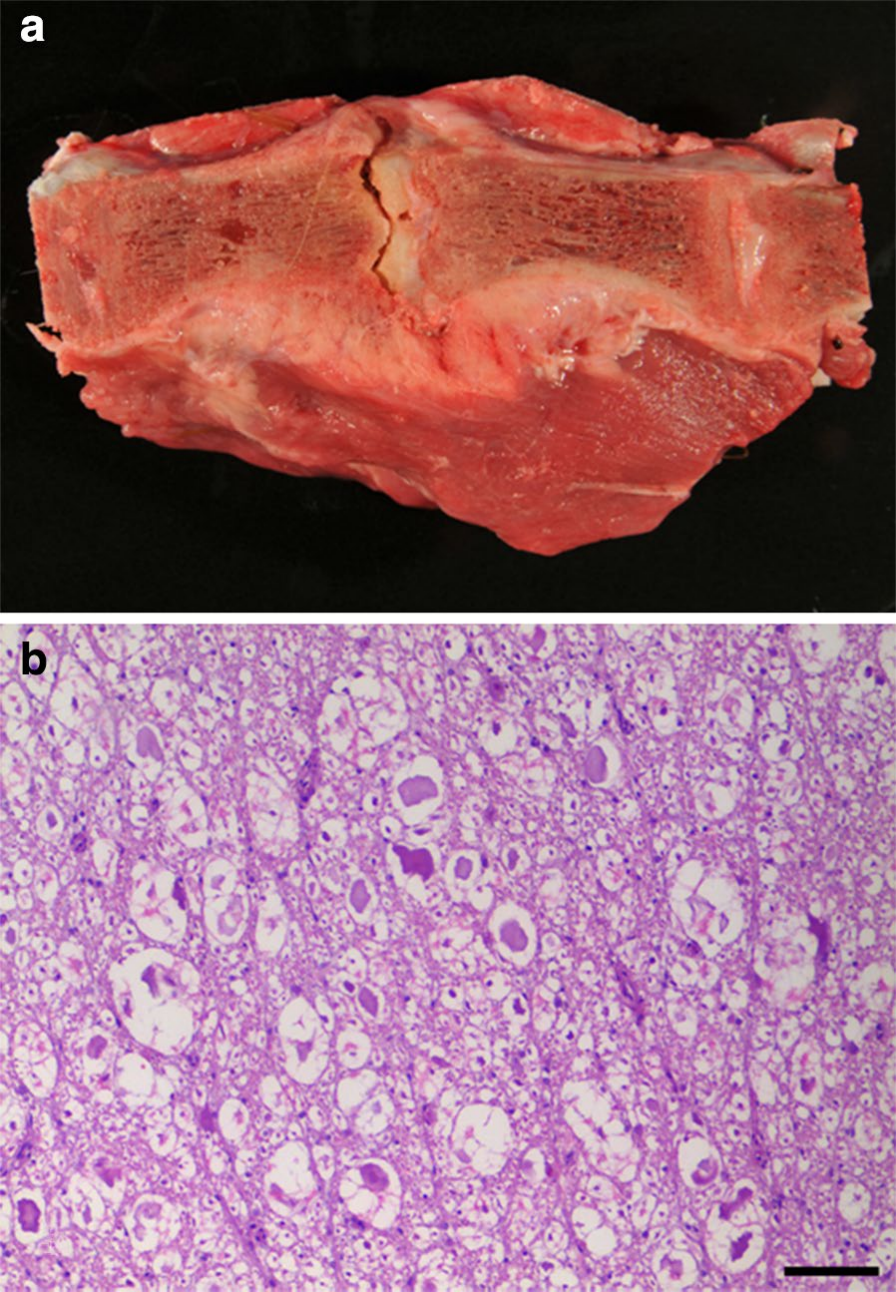
Longitudinal section of the fourth and fifth lumbar vertebrae (L4–L5) of a male alpaca (*Vicugna pacos*). **a** L4 and L5 with severe narrowing of the spinal canal and ventral spondylosis. **b** Histologic section of the L4–L5 spinal cord with *white* matter showing multiple swollen and degenerated axons (*spheroids*) (hematoxylin and eosin; *bar* = 100 μm)

Histopathology revealed massive infiltration of neutrophils and plasma cells and, as well as some macrophages and lymphocytes admixed with fibrin and accumulations of rod-shaped and coccoid bacteria within the intervertebral space, dorsally displacing the degenerated intervertebral disc. The meninges and surrounding adipose tissue of the corresponding section of the lumbar spinal cord showed severe focal infiltration with neutrophils and some plasma cells, lymphocytes, and macrophages, consistent with purulent meningitis. Within the white matter, numerous swollen and degenerated axons (spheroids) were found as a result of the focal compression. These findings were consistent with severe, chronic, purulent discospondylitis (Fig. 2a, b).

Bacterial culture of the spinal cord and the inflamed surrounding tissue of vertebrae L4 and L5 revealed high to moderate numbers of α and γ hemolytic *Streptococcus* spp., *Staphylococcus epidermidis*, and *Escherichia coli*.

## Conclusions

Urolithiasis, chronic renal diseases (i.e., amyloidosis and glomerulonephritis), and acute renal failure have been described in new world camelids [1, 2]. Micturition disorders in camelids can be seen with urolithiasis as well as in acute renal failure [1, 4]. Because the blood urea nitrogen (BUN) and creatinine values of our patient were within the normal ranges, the diagnosis of acute renal failure was excluded. Clinical signs of abdominal discomfort and a distended urinary bladder seen ultrasonographically led to the preliminary diagnosis of urolithiasis. Surgical exploration did not confirm a diagnosis of urolithiasis, and progressive ataxia and reflex deficits led to suspicion of a neurological etiology. In cases of neurological disturbances with impairment of the LMN, a so-called “LMN bladder” may be provoked, wherein ischuria and bladder distension are caused by the detrusor muscle being flaccidly paralyzed [5]. Common causes of LMN impairment are congenital anomalies or traumatic, neoplastic, or infectious lesions of the lumbosacral spinal cord or the hypogastric, pelvic, or pudendal nerve [5, 6]. Normal urination is a complex process of cholinergic (muscarinic and nicotinic receptors) and adrenergic pathways (α- and β-adrenergic receptors) that results in precise interaction of the contraction of the detrusor muscle of the urinary bladder and simultaneous relaxation of the urethral sphincter muscles. Neurologic examination of the alpaca revealed hyporeflexia and decreased muscle tone of the hind limbs. This paresis of the skeletal muscles of the hind limbs of the alpaca, in combination with the distended urinary bladder and the MR images, confirmed the suspicion of a neurological etiology.

Reports of neurological disease in new world camelids are limited [7]. However, ataxia and paresis in llamas and alpacas have been described due to systemic infectious diseases (e.g., equine herpes virus 1 infection, listeriosis), local bacterial infections with discospondylitis, parasite-induced leucomyelopathy, degenerative myeloencephalopathy, or intervertebral disc protrusion [1, 7–10]. In general, discospondylitis seems to be a rather rare disease in new world camelids, with clinical signs similar to those seen in other species but displaying huge variability [9, 11–16]. Depending on the localization of a spinal cord lesion, affected animals show bilateral ataxia, paresis or paralysis. With LMN disease, animals may show decreased or absent reflexes. With UMN disease, normal or exaggerated reflexes with loss of muscle tone or increased muscle tone, respectively, are seen [9]. This alpaca displayed clinical signs typical for lesion localization in segment L4-S5 of the spinal cord, such as recumbency, bilaterally reduced reflexes of the hind limbs with unaffected fore limbs and cranial nerves, and normal mentation [9]. MRI revealed lumbar discospondylitis of L4-L5 with herniation of intervertebral disc material causing compression of 50 % of the spinal cord and the resultant clinical signs. A retrospective study of dogs [12] described discospondylitis due to hematogenous spread of bacteria or iatrogenic etiologies caused by epidural injections. A previous report of discospondylitis in an alpaca failed to identify the bacteriologic agent [15]. However, in this case, α and γ hemolytic streptococci, *S. epidermidis*, and *E. coli* were confirmed by bacterial culture from L4/L5, the spinal cord, and the kidney. These bacteria are consistent with those found in cases of discospondylitis in other species [2] and indicate that the origin of discospondylitis in new world camelids may be similar to that of small animals, even if no specific causes have yet been described for this species [15]. Urogenital infections, abscesses, wound infection leading to bacteremia, respiratory diseases, and infections of the oral cavity have been identified as common causes of discospondylitis in dogs [12]. The owner mentioned that fights between the males of the herd were not infrequent, so trauma could not be ruled out. However, hemorrhage or other gross signs of trauma were not observed in the spine or surrounding soft tissues.

In summary, discospondylitis should be included as a differential diagnosis for ischuria in camelids. Clinical signs of trickling urination in combination with ataxia or delayed reflexes should encourage the veterinarian to perform a detailed neurological examination. Ancillary diagnostics should consist of blood work, ultrasonography, myelography, and/or MRI. In this study, MRI with application of the contrast medium Dotarem ^®^ (gadoteric acid) was used to achieve confirmation of our suspected diagnosis. However, use of this contrast medium is not allowed for use in animals that are intended for human consumption, which limits its use. Nevertheless, national legislation may allow the use of such substances if the animal is securely excluded from the food chain (“declaration of use”; available online, http://www.vetidata.de). Use of Dotarem^®^ in this study was done according to German legislation. In this case, the use of the animal as food producing animal was additionally excluded because of the owner’s decision to euthanize it. The exceptional use of drugs in camelids as well as the prohibitive costs have prevented widespread use of MRI as a diagnostic modality in livestock; therefore, limited data are available. More data are needed to thoroughly describe normal anatomy and pathological conditions of spinal disease in this species.

## References

[1] Kingston JK, Stäempfli HR (1995). Silica urolithiasis in a male llama. Can Vet J.

[2] Smith JA (1989). Noninfectious diseases, metabolic diseases, toxicities, and neoplastic diseases of South American camelids. Vet Clin North Am Food Animal Pract.

[3] Grauer GF, Nelson RW, Couto CG (2010). Diseases of the urinary tract [In German]. Innere Medizin der Kleintiere [Small animal internal medicine].

[4] Gerspach C, Bateman S, Sherding R, Chew DJ, Besier AS, Grieves JL, Lakritz J (2010). Acute renal failure and anuria associated with Vitamin D intoxication in two alpaca (*Vicugna pacos*) cria. J Vet Intern Med.

[5] Dewey CW, Dewey CW (2008). Neurology and neuropharmacology of normal and abnormal urination. A practical guide to canine and feline neurology.

[6] Carr EA, Dawson Soto DR, Smith BP (2015). Urinary incontinence. Large animal internal medicine.

[7] Valentine BA, Saulez MN, Cebra CK, Fischer KA (2006). Compressive myelopathy due to intervertebral disk extrusion in a llama (*Lama glama*). J Vet Diagn Invest.

[8] Barnett JEF, Preston GD, Steele LM, Gibbons LM, Scholes SFE, Schock A, Higgins RJ (2008). Parasite-induced leucomyelopathy in llamas (*Lama glama*). Vet Rec.

[9] Whitehead CE, Bedenice D (2009). Neurologic diseases in llamas and alpacas. Vet Clin Food Anim.

[10] Morin DE, Toenniessen JG, French RA, Knight BL, Zachary JF (1994). Degenerative myeloencephalopathy in two llamas. J Am Vet Med Assoc.

[11] Sweers L, Carstens A (2006). Imaging features of discospondylitis in two horses. Vet Radiol Ultrasound.

[12] Burkert BA, Kerwin SC, Hosgood GL, Pechman RD, Fontenelle JP (2005). Signalment and clinical features of discospondylitis in dogs: 513 cases (1980–2001). J Am Vet Med Assoc.

[13] Muggli E, Schmid T, Hagen R, Schmid B, Nuss K (2011). Diagnosis and treatment of lumbosacral discospondylitis in a calf. Vet Res.

[14] Doige CE (1980). Discospondylitis in swine. Can J Comp Med.

[15] Zanolari P, Konar M, Tomek A, Hoby S, Meylan M (2006). Paraparesis in an adult alpaca with discospondylitis. J Vet Intern Med.

[16] Sura R, Creden A, Van Kruiningen HJ (2008). *Pseudomonas*-associated discospondylitis in a two-month-old llama. J Vet Diagn Invest.

